# Condyloma acuminatum of the urinary bladder with underlying squamous cell carcinoma: a case report

**DOI:** 10.1186/s13256-022-03669-0

**Published:** 2022-12-01

**Authors:** John A. Whitaker

**Affiliations:** 1 School of Medicine, University of Mississippi Medical Center, Jackson, MS USA; 2 Department of Urology, University of Mississippi Medical Center, Jackson, MS USA; 3 Department of General Surgery, Wake Forest University, Winston-Salem, USA

**Keywords:** Condyloma acuminatum, Squamous cell carcinoma, Urinary bladder, Risk factor

## Abstract

**Background:**

Condyloma acuminatum is a rare finding of the urinary bladder. There are many morphological variants that exist. It has been previously reported that some of these variants were found to be associated with subsequent or concurrent squamous cell carcinoma. However, there are limited cases that describe this underlying malignancy found in patients with bladder condyloma.

**Case presentation:**

A 38-year-old African American female presented with condyloma acuminatum of the urinary bladder and underlying squamous cell carcinoma, which also invaded the neighboring uterus, urethra, and anterior vagina. Initial attempts at treatment began with cystoscopic resection of the condyloma; however, due to diffuse transformation, we pursued radical cystectomy with adjuvant chemotherapy and radiation.

**Conclusion:**

As such a finding is rare in the urinary bladder, with few reports discussing its association with ensuing squamous cell carcinoma, we hope that this continues to generate awareness and consideration in the treatment of affected individuals.

## Background

Condyloma acuminatum is an epidermal manifestation that primarily affects the anal and genital regions. Only rarely has it been found outside of these normal parameters in mucocutaneous regions such as the urethra. In some populations, literature has described that condyloma acuminatum act as a risk factor for malignancy. Limited reports describe these lesions associated with squamous cell carcinoma found in the urinary bladder.

## Case presentation

A 38-year-old African American female with no significant past medical history presented to the emergency department as a referral for evaluation in May 2020 owing to a large bladder mass found incidentally on imaging at an outside hospital. At the time of presentation, her only symptoms were recurrent urinary tract infections, of which she reported 4–5 occurrences in the last year, intermittent spotting on toilet paper, and occasional stress urinary incontinence. We repeated a computed tomography (CT) of the abdomen and pelvis at our institution and identified the mass occupying much of the posterior bladder wall with suspicious but indeterminate invasion of the uterus and cervix. Additionally, bilateral hydroureteronephrosis was found with the left ureter more dilated than the right. Subsequently, the patient underwent cystoscopy with bilateral stent placement and a biopsy sample was taken of the bladder lesion as well as a lesion of the left labia. Pathology results returned for both showing findings consistent with condyloma acuminatum as well as low-grade squamous intraepithelial lesion of the cervix. The patient then proceeded with a transurethral resection (TUR) of the mass. A second TUR with excision of the left labial mass was planned owing to the remaining tumor burden and concern that there may be an underlying malignancy. This attempt, however, was aborted owing to diffuse transformation now involving the urethra. After careful consideration and discussion, it was determined that bladder salvage was not feasible, and the best course of action would be bladder removal. She then underwent a radical cystectomy with the creation of a neobladder and self-catheterizable stoma and a hysterectomy in early September 2020, with biopsy samples taken intraoperatively. Final pathology results returned showing atypical condyloma proliferation of the urinary bladder wall and squamous cell carcinoma of the urinary bladder, urethra, and anterior vagina. Staging of the malignancy was determined to be IIIA (pT4a, pN0, cM0) and grade 1 owing to microscopic residual tumor margins of the urethra, as the condyloma was found to extend beyond the urethral meatus. Considering the staging reflected positive margins, adjuvant chemotherapy was carefully considered. She then began chemo and radiation in November 2020 and completed it in February 2021. Today, she is managed with close monitoring by surveillance imaging with urology and medical and radiation oncology with no recurrence of disease demonstrated on imaging.

**Table:** 

Transverse CT abdomen-pelvis showing posterior bladder mass	
Coronal CT abdomen-pelvis showing bladder mass and hydroureteronephrosis	
Sagittal CT abdomen-pelvis showing posterior bladder mass	
Bladder cystoscopy showing condyloma acuminatum	

## Discussion

Condyloma acuminatum occurs as epidermal verrucous growths that primarily affect the genital and anal regions. It is transmitted by sexual contact through human papillomavirus types 6 and 11. Histologically, it appears as hyperplastic papillary fronds with koilocytosis and viral atypia. Whereas it is chiefly found affecting external genitalia, it is less commonly found in mucocutaneous regions such as the urethra [1]. In addition to HPV exposure, and prior anogenital warts, immunosuppressed populations have been found at greater risk of developing condyloma acuminatum, with a higher burden to include treatment resistance, higher rates of recurrence, and higher rates of malignant potential [2, 3]. Rarely, these have been described as noninvasive lesions found in the urinary bladder with few reports recorded in the literature [4].

As one of many morphologic variants found uncommonly in biopsies of the urinary bladder, condyloma acuminatum has been found associated with subsequent or concurrent carcinoma in situ (CIS) or invasive squamous cell carcinoma (SCC) of the bladder. In fact, a clinicopathological analysis of 29 cases involving noninvasive lesions reports their findings of malignant transformations. Of three patients with condyloma acuminatum of the urinary bladder, one patient had CIS at the time of cystectomy at a 3-month interval from biopsy, while another had invasive SCC at 20 months from the time of biopsy [5]. Another study showed that 17 of 38 patients with invasive SCC or CIS were diagnosed concurrently or within 1 year of being found to have condyloma acuminatum of the urinary bladder. Therefore, though limited data exist owing to few reported incidences, documented discoveries suggest that condyloma acuminatum of the urinary tract increases the risk for bladder squamous cell carcinoma.

Most bladder cancers are urothelial in nature, with less than 5% of bladder cancers being non-urothelial in nature [6]. These non-urothelial bladder cancers represent up to 75% of bladder malignancy in areas where *Schistosoma hematobium* infection is endemic [7]. While several risk factors have been shown to be associated with bladder cancer, environmental exposures to things such as cigarette smoke remain chiefly responsible [8, 9]. It should be noted, however, that one study even found a 50% increase in the risk of overall cancer among patients hospitalized with condyloma acuminatum [10]. This is especially concerning when considering shared risk factors of both condyloma acuminatum and bladder cancer such as smoking [11, 12].

Human papillomavirus continues to represent the most common sexually transmitted infection worldwide and predisposes affected individuals to condyloma acuminatum [3]. As condyloma acuminatum has been found to be associated with developing concurrent or subsequent SCC of the bladder, clinicians should take special consideration when exploring squamous lesions of the bladder, as these lesions can represent underlying malignancy. This is especially true when considering the significant morphological overlap between various noninvasive squamous lesions of the bladder, as these can make diagnosing condyloma acuminatum difficult [4]. With bladder cancer in the USA steadily increasing, special attention should be given to immunocompromised individuals, as they have been found to be associated with an increased risk of developing condyloma acuminatum and subsequently SCC of the bladder [2, 3]. Condyloma acuminatum has been previously documented in post-transplant patients, diabetics, and those with autoimmune diseases such as rheumatoid arthritis [13, 14].

Treatment for condyloma acuminatum of the external genitalia has been widely accepted as either monotherapy or a combination of various medical therapies, although local recurrence is typical [15]. Surgical options vary and range anywhere from cryoablation, laser ablation, electrocautery, or excision. The selection of treatment varies by circumstance and depends on the number, size, and location of lesions. Thus, it is determined on a case-by-case basis. As a rare finding in the urinary bladder, little evidence is available for the standard of care and guiding treatment approach. In general, trends in practices start with conservative measures but escalate to radical surgical treatment upon the findings of malignant transformation [16], though, one study reports successful treatment with radiation and low-dose chemo without cystectomy in patients with condyloma alone [17].

Treatment of squamous cell carcinoma of the bladder is chiefly radical resection of the urinary bladder [18, 19]. Evidence from retrospective and observational data shows that squamous cell carcinoma appears to be more aggressive than urothelial carcinoma [20]. While there is no defined role for neoadjuvant or adjuvant radiation therapy, certain patients may benefit especially in the context of persistent local advanced disease despite cystectomy [21]. In patients with advanced bladder cancer where surgery is not an option, palliative care is a reasonable approach. Still, with limited prospective data, treatment options should be pursued on an individual basis. As it stands, the prognosis is difficult to calculate. However, one multi-institutional study reports no difference in 5-year survival between urothelial and non-urothelial bladder cancers following radical cystectomy after controlling for sex, stage, and grade [22].

## Conclusion

We describe a rare case in which condyloma acuminatum of the urinary bladder was found, with final pathology results revealing underlying SCC of the bladder. Though it is rarely reported, condyloma acuminatum represents one of a few noninvasive squamous lesions of the urinary bladder. Immunocompromised patients appear to be at a greater risk of developing condyloma acuminatum, and in this population, higher reports of disease burden have been found. Owing to the rising prevalence of HPV worldwide and the increasing number of reports documenting condyloma of the bladder found in more subtypes of immunocompromised individuals, it is of growing urological concern. In general, limited data are available to guide treatment options for these skin lesions; however, it is common to pursue conservative measures and escalate to radical surgical options depending on the tumor burden. Condyloma acuminatum has been found to be associated with subsequent or concurrent CIS or SCC of the bladder upon initial biopsy determination. As such, patients found with condyloma of the bladder should be closely monitored for underlying malignancy. Treatment usually involves radical cystectomy, though preoperative or postoperative combinations of chemotherapy or radiation are considered in certain patient populations. As a rare finding, the prognosis remains to be determined.

## Data Availability

Not applicable.

## Abbreviations

SCC: Squamous cell carcinoma
TUR: Transurethral resection
HPV: Human Papillomavirus
CIS: Carcinoma in situ
